# 2-(4-Methyl­anilino)acetohydrazide

**DOI:** 10.1107/S1600536809033169

**Published:** 2009-08-26

**Authors:** Hoong-Kun Fun, Chin Sing Yeap, Shridhar Malladi, Mahesh Padaki, Arun M. Isloor

**Affiliations:** a X-ray Crystallography Unit, School of Physics, Universiti Sains Malaysia, 11800 USM, Penang, Malaysia; b Department of Chemistry, National Institute of Technology–Karnataka, Surathkal, Mangalore 575 025, India

## Abstract

In the title mol­ecule, C_9_H_13_N_3_O, the non-hydrogen atoms of the hydrazide group are essentially planar [maximum deviation = 0.028 (1) Å for one of the N atoms]. The mean plane of this group forms a dihedral angle of 83.34 (5)° with the plane of the benzene ring. In the crystal structure, mol­ecules are linked by inter­molecular N—H⋯O, N—H⋯N and weak C—H⋯N hydrogen bonds into a two-dimensional network parallel to the *ab* plane. Additional stabilization is provided by a weak C—H⋯π inter­action.

## Related literature

For the biological activity of hydrazide derivatives, see: Ozdemir *et al.* (2009 ▶); Khattab (2005 ▶). For synthetic applications, see: Isloor *et al.* (2009 ▶); Holla & Udupa (1992 ▶). For a related structure, see: Zhang & Shi (2009 ▶). For the stability of the temperature controller used for the data collection, see: Cosier & Glazer (1986 ▶).
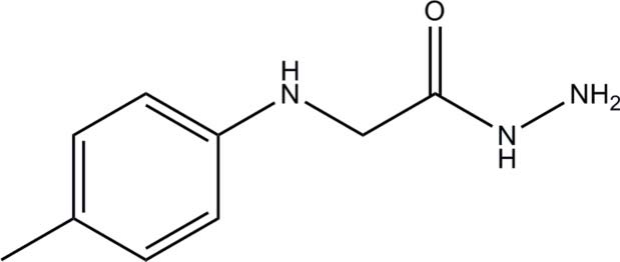


## Experimental

### 

#### Crystal data

C_9_H_13_N_3_O
*M* *_r_* = 179.22
Triclinic, 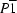

*a* = 5.1481 (1) Å
*b* = 5.9262 (2) Å
*c* = 15.4756 (4) Å
α = 87.002 (2)°
β = 84.282 (2)°
γ = 82.849 (2)°
*V* = 465.76 (2) Å^3^
*Z* = 2
Mo *K*α radiation
μ = 0.09 mm^−1^
*T* = 100 K
0.52 × 0.15 × 0.07 mm

#### Data collection

Bruker SMART APEXII CCD area-detector diffractometer
Absorption correction: multi-scan (**SADABS**; Bruker, 2005 ▶) *T*_min_ = 0.956, *T*_max_ = 0.994
11697 measured reflections
2703 independent reflections
2301 reflections with *I* > 2σ(*I*)
*R*_int_ = 0.026

#### Refinement

*R*[*F* ^2^ > 2σ(*F* ^2^)] = 0.039
*wR*(*F* ^2^) = 0.110
*S* = 1.03
2703 reflections
170 parameters
H atoms treated by a mixture of independent and constrained refinement
Δρ_max_ = 0.34 e Å^−3^
Δρ_min_ = −0.20 e Å^−3^

### 

Data collection: *APEX2* (Bruker, 2005 ▶); cell refinement: *SAINT* (Bruker, 2005 ▶); data reduction: *SAINT*; program(s) used to solve structure: *SHELXTL* (Sheldrick, 2008 ▶); program(s) used to refine structure: *SHELXTL*; molecular graphics: *SHELXTL*; software used to prepare material for publication: *SHELXTL* and *PLATON* (Spek, 2009 ▶).

## Supplementary Material

Crystal structure: contains datablocks global, I. DOI: 10.1107/S1600536809033169/lh2884sup1.cif

Structure factors: contains datablocks I. DOI: 10.1107/S1600536809033169/lh2884Isup2.hkl

Additional supplementary materials:  crystallographic information; 3D view; checkCIF report

## Figures and Tables

**Table 1:** Hydrogen-bond geometry (Å, °)

*D*—H⋯*A*	*D*—H	H⋯*A*	*D*⋯*A*	*D*—H⋯*A*
N1—H1*N*1⋯O1^i^	0.875 (17)	2.162 (17)	3.0271 (11)	170.0 (13)
N2—H1*N*2⋯N3^ii^	0.887 (17)	2.287 (16)	3.0302 (12)	141.3 (13)
N3—H1*N*3⋯O1^iii^	0.901 (14)	2.252 (13)	3.0614 (11)	149.4 (13)
N3—H2*N*3⋯O1^iv^	0.914 (14)	2.281 (15)	3.0889 (12)	147.1 (12)
C7—H7*B*⋯N3^v^	0.991 (15)	2.545 (14)	3.4341 (14)	149.2 (11)
C9—H9*B*⋯*Cg*^iv^	0.96 (2)	2.94 (2)	3.7469 (16)	142.3 (14)

Symmetry codes: (i) 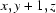; (ii) 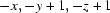; (iii) 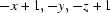; (iv) 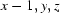; (v) 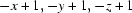. *Cg* is the centroid of the C1–C6 benzene ring.

**Fig. 1.**
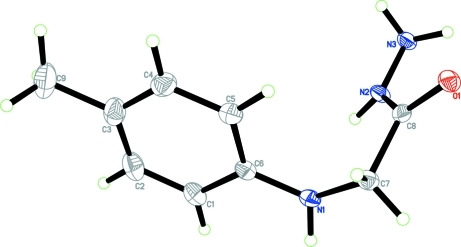
The molecular structure of the title compound with atom labels and 50% probability ellipsoids for non-H atoms.

**Fig. 2.**
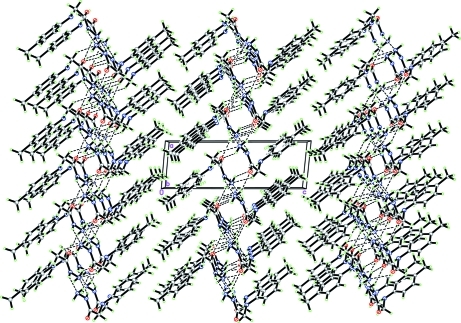
Part of the crystal structure of (I), viewed along the b axis, showing the two-dimensional network parallel to ab plane. Intermolecular hydrogen bonds are shown in as dashed lines.

## supplementary crystallographic information

### Comment

In organic chemistry, hydrazides are a class of organic compounds sharing a common functional group characterized by a nitrogen to nitrogen covalent bond with 4 substituents with at least one of them being an acyl group. They are also starting materials for many heterocycles including 1,2,4-triazoles (Isloor *et al.*, 2009; Holla & Udupa, 1992). Many hydrazide derivatives have showed significant biological activities (Ozdemir *et al.*, 2009; Khattab, 2005). In view of the biological and synthetic importance of hydrazides, we hereby report the crystal structure of the title compound (I).

The bond lengths and angles of the title compound (I), (Fig. 1) are comparable to its related structure (Zhang & Shi, 2009). A maximum deviation of 0.028 (1) Å for atom N2 from the mean plane of the hydrazide group form by atoms O1, N2, N3, C7 and C8 indicates that it is essentially coplanar. The mean plane of the hydrazide makes dihedral angle of 83.34 (5)° with C1–C6 benzene ring. In the crystal structure, the molecules are linked by intermolecular N1—H1N1···O1^i^, N2—H1N2···N3^ii^, N3—H1N3···O1^iii^, N3—H2N3···O1^iv^ and C7—H7B···N3^v^ (see Table 1 for symmetry codes) hydrogen bonds into two-dimensional network parallel to *ab* plane (Fig. 2, Table 1). The crystal structure is also stabilized by a C—H···π interaction (Table 1).

### Experimental

Ethyl [(4-methylphenyl)amino]acetate (19.3 g, 0.1 mol) and hydrazine hydrate (99%, 0.2 mol) in ethanol (200 ml) was heated on a water-bath for 6 h. Excess of ethanol was removed by distillation. On cooling, colourless block-shaped single crystals of 2-[(4-methylphenyl)amino]acetohydrazide begin to separate (Holla & Udupa, 1992). It was collected by filtration and recrystallized from ethanol. Yield: 13.2 g, 73.7%, *M.p.* 423–426 K.

### Refinement

All hydrogen atoms were located from the difference Fourier map and refined freely, with N—H = 0.876 (15)–0.912 (15) Å; C–H = 0.96 (2)–1.04 (2) Å.

### Crystal data

**Table:**

C_9_H_13_N_3_O	*Z* = 2
*M**_r_* = 179.22	*F*(000) = 192
Triclinic, *P*1	*D*_x_ = 1.278 Mg m^−^^3^
Hall symbol: -P 1	Mo *K*α radiation, λ = 0.71073 Å
*a* = 5.1481 (1) Å	Cell parameters from 6230 reflections
*b* = 5.9262 (2) Å	θ = 2.7–31.2°
*c* = 15.4756 (4) Å	µ = 0.09 mm^−^^1^
α = 87.002 (2)°	*T* = 100 K
β = 84.282 (2)°	Block, colourless
γ = 82.849 (2)°	0.52 × 0.15 × 0.07 mm
*V* = 465.76 (2) Å^3^	

### Data collection

**Table:**

Bruker SMART APEXII CCD area-detector diffractometer	2703 independent reflections
Radiation source: fine-focus sealed tube	2301 reflections with *I* > 2σ(*I*)
graphite	*R*_int_ = 0.026
φ and ω scans	θ_max_ = 30.0°, θ_min_ = 2.7°
Absorption correction: multi-scan (*SADABS*; Bruker, 2005)	*h* = −7→7
*T*_min_ = 0.956, *T*_max_ = 0.994	*k* = −8→8
11697 measured reflections	*l* = −21→21

### Refinement

**Table:**

Refinement on *F*^2^	Primary atom site location: structure-invariant direct methods
Least-squares matrix: full	Secondary atom site location: difference Fourier map
*R*[*F*^2^ > 2σ(*F*^2^)] = 0.039	Hydrogen site location: inferred from neighbouring sites
*wR*(*F*^2^) = 0.110	H atoms treated by a mixture of independent and constrained refinement
*S* = 1.03	*w* = 1/[σ^2^(*F*_o_^2^) + (0.0559*P*)^2^ + 0.1344*P*] where *P* = (*F*_o_^2^ + 2*F*_c_^2^)/3
2703 reflections	(Δ/σ)_max_ < 0.001
170 parameters	Δρ_max_ = 0.34 e Å^−^^3^
0 restraints	Δρ_min_ = −0.20 e Å^−^^3^

### Special details

**Table:**

Experimental. The crystal was placed in the cold stream of an Oxford Cyrosystems Cobra open-flow nitrogen cryostat (Cosier & Glazer, 1986) operating at 100.0 (1) K.
Geometry. All e.s.d.'s (except the e.s.d. in the dihedral angle between two l.s. planes) are estimated using the full covariance matrix. The cell e.s.d.'s are taken into account individually in the estimation of e.s.d.'s in distances, angles and torsion angles; correlations between e.s.d.'s in cell parameters are only used when they are defined by crystal symmetry. An approximate (isotropic) treatment of cell e.s.d.'s is used for estimating e.s.d.'s involving l.s. planes.
Refinement. Refinement of *F*^2^ against ALL reflections. The weighted *R*-factor *wR* and goodness of fit *S* are based on *F*^2^, conventional *R*-factors *R* are based on *F*, with *F* set to zero for negative *F*^2^. The threshold expression of *F*^2^ > σ(*F*^2^) is used only for calculating *R*-factors(gt) *etc*. and is not relevant to the choice of reflections for refinement. *R*-factors based on *F*^2^ are statistically about twice as large as those based on *F*, and *R*- factors based on ALL data will be even larger.

### Fractional atomic coordinates and isotropic or equivalent isotropic displacement parameters (Å^2^)

**Table:**

	*x*	*y*	*z*	*U*_iso_*/*U*_eq_	
O1	0.63005 (14)	0.15409 (11)	0.39934 (5)	0.01915 (17)	
N1	0.43188 (17)	0.74133 (14)	0.33320 (6)	0.01783 (18)	
N2	0.26039 (16)	0.39523 (13)	0.43209 (5)	0.01650 (18)	
N3	0.12716 (17)	0.23345 (14)	0.48445 (6)	0.01765 (18)	
C1	0.1124 (2)	0.95874 (17)	0.24809 (7)	0.0231 (2)	
C2	−0.0573 (2)	0.9751 (2)	0.18340 (8)	0.0282 (2)	
C3	−0.0748 (2)	0.7920 (2)	0.13190 (7)	0.0280 (2)	
C4	0.0857 (2)	0.5915 (2)	0.14806 (7)	0.0257 (2)	
C5	0.2577 (2)	0.57064 (17)	0.21309 (7)	0.0208 (2)	
C6	0.27314 (19)	0.75502 (16)	0.26432 (6)	0.01770 (19)	
C7	0.62171 (19)	0.54434 (16)	0.34553 (7)	0.01795 (19)	
C8	0.50373 (18)	0.34635 (15)	0.39460 (6)	0.01517 (18)	
C9	−0.2624 (3)	0.8132 (3)	0.06212 (9)	0.0405 (3)	
H1A	0.116 (3)	1.091 (3)	0.2848 (10)	0.031 (4)*	
H2A	−0.167 (3)	1.117 (3)	0.1751 (11)	0.042 (4)*	
H4A	0.081 (3)	0.457 (3)	0.1131 (10)	0.032 (4)*	
H5A	0.363 (3)	0.424 (3)	0.2235 (10)	0.031 (4)*	
H7A	0.713 (3)	0.484 (2)	0.2916 (9)	0.024 (3)*	
H7B	0.757 (3)	0.592 (2)	0.3797 (9)	0.026 (3)*	
H9A	−0.199 (4)	0.895 (4)	0.0098 (14)	0.070 (6)*	
H9B	−0.435 (4)	0.878 (3)	0.0840 (13)	0.064 (6)*	
H9C	−0.290 (4)	0.655 (4)	0.0400 (14)	0.072 (6)*	
H1N1	0.485 (3)	0.869 (3)	0.3461 (9)	0.029 (4)*	
H1N2	0.185 (3)	0.538 (3)	0.4343 (9)	0.028 (3)*	
H1N3	0.250 (3)	0.125 (2)	0.5030 (9)	0.023 (3)*	
H2N3	0.031 (3)	0.167 (2)	0.4485 (9)	0.028 (4)*	

### Atomic displacement parameters (Å^2^)

**Table:**

	*U*^11^	*U*^22^	*U*^33^	*U*^12^	*U*^13^	*U*^23^
O1	0.0173 (3)	0.0139 (3)	0.0257 (4)	0.0010 (2)	−0.0024 (3)	−0.0012 (3)
N1	0.0208 (4)	0.0112 (3)	0.0219 (4)	−0.0032 (3)	−0.0028 (3)	−0.0005 (3)
N2	0.0155 (4)	0.0110 (3)	0.0223 (4)	−0.0013 (3)	0.0001 (3)	0.0014 (3)
N3	0.0159 (4)	0.0135 (4)	0.0233 (4)	−0.0029 (3)	−0.0015 (3)	0.0035 (3)
C1	0.0247 (5)	0.0183 (5)	0.0246 (5)	0.0002 (4)	0.0004 (4)	0.0021 (4)
C2	0.0246 (5)	0.0294 (6)	0.0277 (6)	0.0031 (4)	−0.0008 (4)	0.0081 (4)
C3	0.0224 (5)	0.0410 (6)	0.0209 (5)	−0.0083 (4)	−0.0023 (4)	0.0072 (4)
C4	0.0274 (5)	0.0303 (5)	0.0210 (5)	−0.0109 (4)	−0.0013 (4)	−0.0011 (4)
C5	0.0225 (5)	0.0183 (4)	0.0216 (5)	−0.0041 (3)	−0.0006 (4)	−0.0007 (3)
C6	0.0178 (4)	0.0160 (4)	0.0189 (4)	−0.0033 (3)	0.0006 (3)	0.0015 (3)
C7	0.0156 (4)	0.0158 (4)	0.0222 (5)	−0.0027 (3)	−0.0004 (3)	0.0010 (3)
C8	0.0156 (4)	0.0140 (4)	0.0165 (4)	−0.0020 (3)	−0.0032 (3)	−0.0019 (3)
C9	0.0291 (7)	0.0679 (10)	0.0258 (6)	−0.0130 (6)	−0.0079 (5)	0.0127 (6)

### Geometric parameters (Å, °)

**Table:**

O1—C8	1.2421 (11)	C2—H2A	0.962 (17)
N1—C6	1.3995 (13)	C3—C4	1.3867 (17)
N1—C7	1.4437 (12)	C3—C9	1.5094 (17)
N1—H1N1	0.876 (15)	C4—C5	1.3961 (15)
N2—C8	1.3313 (12)	C4—H4A	0.988 (15)
N2—N3	1.4205 (11)	C5—C6	1.3978 (14)
N2—H1N2	0.886 (15)	C5—H5A	0.979 (15)
N3—H1N3	0.902 (14)	C7—C8	1.5213 (13)
N3—H2N3	0.912 (15)	C7—H7A	0.978 (14)
C1—C2	1.3850 (16)	C7—H7B	0.989 (14)
C1—C6	1.4022 (13)	C9—H9A	0.97 (2)
C1—H1A	0.993 (15)	C9—H9B	0.96 (2)
C2—C3	1.3963 (18)	C9—H9C	1.04 (2)
			
C6—N1—C7	120.37 (8)	C4—C5—C6	120.25 (10)
C6—N1—H1N1	115.9 (10)	C4—C5—H5A	119.3 (9)
C7—N1—H1N1	113.7 (10)	C6—C5—H5A	120.4 (9)
C8—N2—N3	122.60 (8)	C5—C6—N1	122.90 (9)
C8—N2—H1N2	120.9 (10)	C5—C6—C1	118.06 (10)
N3—N2—H1N2	115.5 (10)	N1—C6—C1	118.97 (9)
N2—N3—H1N3	107.5 (9)	N1—C7—C8	113.36 (8)
N2—N3—H2N3	106.4 (9)	N1—C7—H7A	114.3 (8)
H1N3—N3—H2N3	107.4 (12)	C8—C7—H7A	106.5 (8)
C2—C1—C6	120.65 (10)	N1—C7—H7B	107.1 (8)
C2—C1—H1A	119.7 (9)	C8—C7—H7B	108.4 (8)
C6—C1—H1A	119.6 (9)	H7A—C7—H7B	107.0 (12)
C1—C2—C3	121.82 (10)	O1—C8—N2	123.22 (9)
C1—C2—H2A	118.2 (10)	O1—C8—C7	121.37 (9)
C3—C2—H2A	120.0 (10)	N2—C8—C7	115.40 (8)
C4—C3—C2	117.19 (10)	C3—C9—H9A	113.1 (13)
C4—C3—C9	121.88 (12)	C3—C9—H9B	111.3 (12)
C2—C3—C9	120.92 (12)	H9A—C9—H9B	111.5 (17)
C3—C4—C5	122.03 (10)	C3—C9—H9C	112.3 (12)
C3—C4—H4A	120.2 (9)	H9A—C9—H9C	103.7 (17)
C5—C4—H4A	117.8 (9)	H9B—C9—H9C	104.4 (17)
			
C6—C1—C2—C3	−0.26 (17)	C7—N1—C6—C1	−172.79 (9)
C1—C2—C3—C4	−0.06 (17)	C2—C1—C6—C5	0.35 (15)
C1—C2—C3—C9	179.61 (11)	C2—C1—C6—N1	−176.67 (9)
C2—C3—C4—C5	0.28 (16)	C6—N1—C7—C8	−83.08 (11)
C9—C3—C4—C5	−179.38 (11)	N3—N2—C8—O1	2.92 (14)
C3—C4—C5—C6	−0.19 (16)	N3—N2—C8—C7	−176.34 (8)
C4—C5—C6—N1	176.77 (9)	N1—C7—C8—O1	169.09 (8)
C4—C5—C6—C1	−0.13 (15)	N1—C7—C8—N2	−11.63 (12)
C7—N1—C6—C5	10.34 (14)		

### Hydrogen-bond geometry (Å, °)

**Table:**

*D*—H···*A*	*D*—H	H···*A*	*D*···*A*	*D*—H···*A*
N1—H1N1···O1^i^	0.875 (17)	2.162 (17)	3.0271 (11)	170.0 (13)
N2—H1N2···N3^ii^	0.887 (17)	2.287 (16)	3.0302 (12)	141.3 (13)
N3—H1N3···O1^iii^	0.901 (14)	2.252 (13)	3.0614 (11)	149.4 (13)
N3—H2N3···O1^iv^	0.914 (14)	2.281 (15)	3.0889 (12)	147.1 (12)
C7—H7B···N3^v^	0.991 (15)	2.545 (14)	3.4341 (14)	149.2 (11)
C9—H9B···Cg^iv^	0.96 (2)	2.94 (2)	3.7469 (16)	142.3 (14)

Symmetry codes: (i) *x*, *y*+1, *z*; (ii) −*x*, −*y*+1, −*z*+1; (iii) −*x*+1, −*y*, −*z*+1; (iv) *x*−1, *y*, *z*; (v) −*x*+1, −*y*+1, −*z*+1.
